# Genomic insight into the diversity of *Glaesserella parasuis* isolates from 19 countries

**DOI:** 10.1128/msphere.00231-24

**Published:** 2024-08-28

**Authors:** Xiaowei Gong, Qingpo Cui, Wanjiang Zhang, Yuqian Shi, Peng Zhang, Chaoyang Zhang, Gongzheng Hu, Orhan Sahin, Lu Wang, Zhangqi Shen, Mengjiao Fu

**Affiliations:** 1 National Key Laboratory of Veterinary Public Health and Safety, College of Veterinary Medicine, China Agricultural University, Beijing, China; 2 Beijing Key Laboratory of Detection Technology for Animal-Derived Food Safety, College of Veterinary Medicine, China Agricultural University, Beijing, China; 3 State Key Laboratory of Veterinary Biotechnology, Harbin Veterinary Research Institute, Chinese Academy of Agricultural Sciences, Harbin, Heilongjiang, China; 4 College of Veterinary Medicine, Henan Agricultural University, Zhengzhou, Henan, China; 5 Department of Veterinary Diagnostic and Production Animal Medicine, Iowa State University, Ames, Iowa, USA; University of Napoli Federico II, Naples, Italy

**Keywords:** *Glaesserella parasuis*, sequence type, serovar, antimicrobial resistance gene, virulence factor

## Abstract

*Glaesserella parasuis* is a commensal bacterial organism found in the upper respiratory tract of healthy pigs and the etiological agent of Glässer’s disease, which causes severe economic losses in the swine industry. This study aimed to better understand the epidemiological characteristics of this opportunistic pathogen. We investigated the prevalence and distribution of sequence types (STs), serovars, antimicrobial resistance genes (ARGs), and potential virulence factors (VFs) in 764 *G*. *parasuis* isolates collected from diseased and healthy pigs from 19 countries, including China. Multilocus sequence typing showed a high degree of variation with 334 STs, of which 93 were not previously recognized. Phylogenetic analysis revealed two major clades distinguished by isolation year, source, country, and serovar. The dominant serovars of *G. parasuis* were serovars 4 (19.50%), 7 (15.97%), 5/12 (13.87%), and 13 (12.30%). Serovar 7 gradually became one of the dominant serovars in *G. parasuis* with more VFs and fewer ARGs. Serovars 4 and 5/12 were the most frequent serovars in diseased pigs, whereas serovars 2, 8, and 11 were predominant in healthy pigs. Serovars 7 and 13 possessed more VFs than the other serovars. This study provides novel insights into the global prevalence and epidemiology of *G. parasuis* and valuable clues for further investigation into the pathogenicity of *G. parasuis*, which will facilitate the development of effective vaccines.

**IMPORTANCE:**

*Glaesserella parasuis* is a clinically important gram-negative opportunistic pathogen, which causes serious financial losses in swine industry on a global scale. No vaccine is known that provides cross-protection against all 15 serovars; furthermore, the correlation between serovar and virulence is largely unknown. This study provides a large number of sequenced strains in 19 countries and compares the genomic diversity of *G. parasuis* between diseased and healthy pigs. We found a slight change in the dominant serovar of *G. parasuis* in the world, with serovar 7 gradually emerging as one of the predominant serovars. The observed higher average number of VFs in this particular serovar strain challenges the previously held notion that serovar 7 is non-virulent, indicating a more complex virulence landscape than previously understood. Our analysis indicating that six ARGs [*tet*(B), *sul2*, *aph(3')-Ia*, *aph (6)-Id*, *bla*_ROB-1_, and *aph(3'')-Ib*] are likely to be transmitted horizontally in their entirety. By analyzing VFs, we provided an improved understanding of the virulence of *G. parasuis*, and these key findings suggest that vaccine development will be challenging.

## INTRODUCTION

*Glaesserella parasuis* is a gram-negative bacterium of the family Pasteurellaceae and one of the most important conditional pathogens in the modern swine industry (1). It is the etiological agent of Glässer’s disease, which leads to pneumonia, polyserositis, meningitis, and arthritis, causing considerable economic losses to the global swine industry (2). *G. parasuis* commonly colonizes the upper respiratory tract of healthy pigs as a commensal organism. However, it can cause clinical infections under specific circumstances, such as stress, immunosuppression, and poor management (3, 4). Clinically, *G. parasuis* coinfection occurs frequently with other viral and bacterial respiratory pathogens in pigs, such as porcine reproductive and respiratory syndrome virus, porcine circovirus type 2, swine influenza virus, *Streptococcus suis*, *Actinobacillus pleuropneumoniae*, *Pasteurella multocida*, *Bordetella bronchiseptica*, and *Mycoplasma hyopneumoniae*, leading to increased morbidity and mortality in the swine industry worldwide (5–8). Vaccination is an effective strategy for preventing *G. parasuis* infection. However, the absence of commercially available cross-protective vaccines commonly leads to the failure of protection, as reviewed by Macedo et al. (9). Antimicrobial agents are widely used to prevent and control *G. parasuis* infection. However, the global rise in antimicrobial resistance and multidrug resistance makes many commonly used antibiotics ineffective, resulting in more severe challenges to the control of *G. parasuis* infection.

To date, *G. parasuis* has been classified into at least 15 different serovars by traditional gel immunodiffusion assay, indirect hemagglutination, and multiplex PCR assays (the latter cannot distinguish between serovars 5 and 12) (10–12). However, there are still many non-typeable (NT) isolates, ranging from 10% to 41%, even with the serotyping methods described above (13–15). The 15 serovars possess varying levels of virulence; typically, serovars 1, 5, 10, 12, 13, and 14 are considered highly virulent; serovars 2, 4, 8, and 15 are regarded as moderately virulent; and serovars 3, 6, 7, 9, and 11 are deemed as non-virulent (10). However, strains with the same serovar may display different degrees of virulence; for example, strains of serovars 10 and 12 presenting highly virulent do not always produce disease or only cause mild disease, and strains of serovar 7 presenting non-virulent can produce severe disease and classical signs of Glässer’s disease occasionally (16, 17). These studies indicate that the serovar is not a reliable indicator of virulence in *G. parasuis*.

Although the pathogenesis of *G. parasuis* remains poorly understood, an increasing number of pathogenic mechanisms and virulence-associated genes have been identified. The virulence of *G. parasuis* is associated with the evasion of innate immunity by degradation of IgA, resistance to phagocytosis by macrophages, and resistance to killing by complement (18–20). Thus, *G. parasuis* can colonize and initiate infection by adhering to and invading host epithelial and endothelial cells (21, 22). The potential virulence-associated factors have been identified in *G. parasuis*, including capsule, lipopolysaccharides, distinct outer membrane proteins, fimbria, hemolysin, trimeric autotransporters, lipooligosaccharide (LOS) sialylation, cytolethal distending toxin, and neuraminidase (2, 19, 23–26). It is worth noting that the virulence-associated trimeric autotransporters (*vtaA*) have already been used to develop molecular methods to predict the virulence potential of *G. parasuis* (27–29). However, the relationship between virulence-associated factors and clinical pathogenicity remains unclear and requires further investigation.

Through the comprehensive analysis of genomic data from whole-genome sequencing (WGS), we can gain insights into the epidemiological distribution of different serovars of *G. parasuis* both in China and globally. It is crucial for the development of vaccines offering cross-protection. Furthermore, evaluating the carriage of ARGs can provide a basis for clinical medication and facilitate the surveillance of the emergence of multidrug resistance, even resistance to clinical first-line drugs. Genomic analysis also enables the identification of VFs associated with clinical pathogenicity of *G. parasuis*, offering targets for investigating the pathogenic mechanisms and developing attenuated vaccines, thereby contributing to the prevention and control of *G. parasuis* infection. To the best of our knowledge, there have been no studies on the genomic diversity of a large number of *G. parasuis* species. In particular, comparative studies on isolates between diseased and healthy pigs are lacking. This study aimed to gain insight into the structural organization of genetic information and evolutionary relationships among 764 strains of *G. parasuis* collected from 19 countries. WGS analysis was used to determine the distribution of serovars, STs, ARGs, and potential VFs. Moreover, correlations between serovars, ARGs, and VFs were investigated to provide information for better control of *G. parasuis* infection in swine production.

## RESULTS

### Composition and distribution of 764 *G. parasuis*

**Fig 1:**
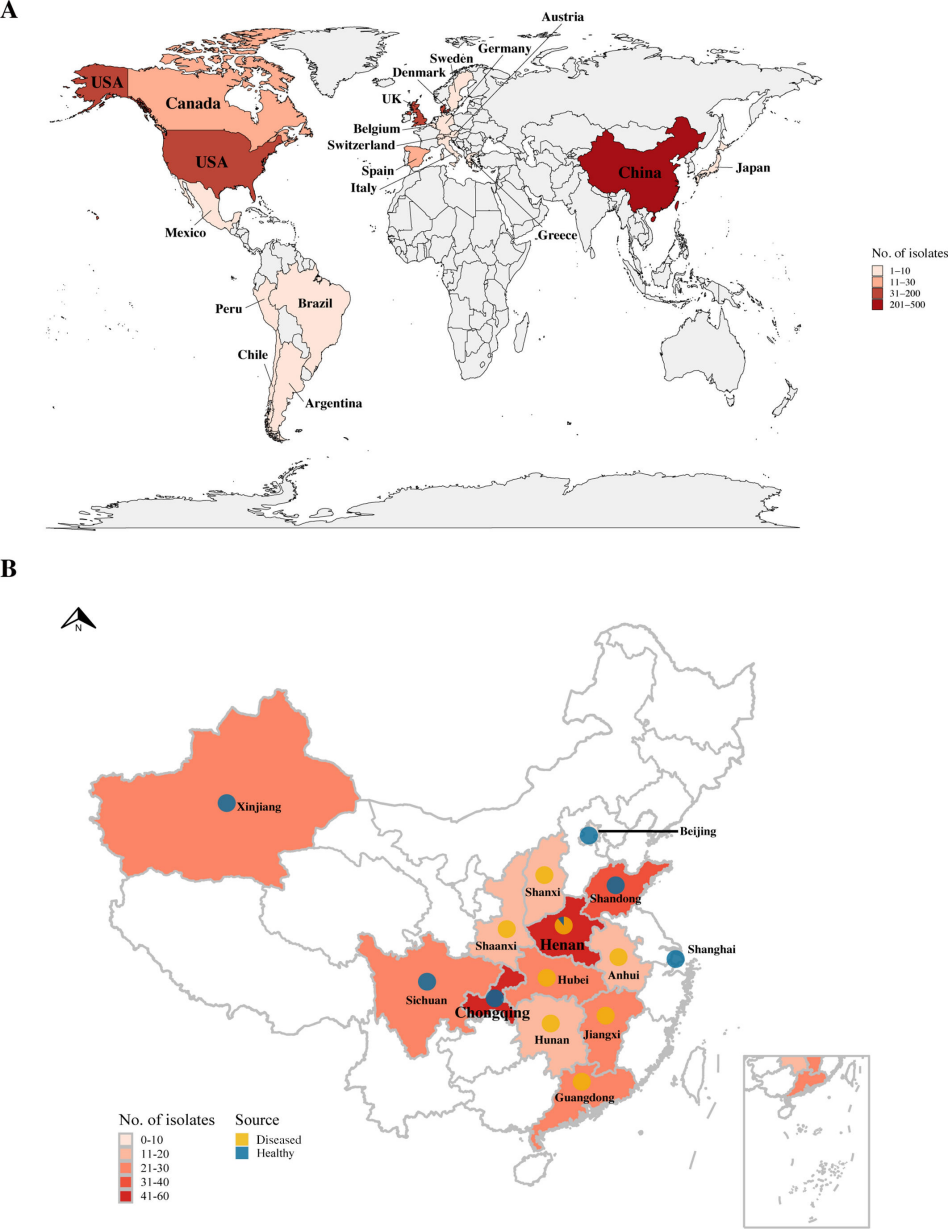
Geographic distribution of 764 *G*. *parasuis* isolates. (**A**) Geographic distribution of 764 *G*. *parasuis* isolates. (**B**) Distribution of 342 *G*. *parasuis* isolates from 14 PLADs in China. The pie charts show the different sources of the isolates. Yellow indicates the isolates collected from diseased pigs; blue indicates the isolates collected from healthy pigs.

In total, 764 genomes [342 strains assembled in this study and 422 genomes from the National Center for Biotechnology Information (NCBI) and European Nucleotide Archive (ENA) database as of December 2022] originating from 19 countries were included in this study (Fig. 1A). Most isolates originated from China (*n* = 422), the USA (*n* = 131), and the UK (*n* = 102), followed by Denmark (*n* = 30), Spain (*n* = 20), Canada (*n* = 19), Japan (*n* = 8), Chile (*n* = 7), Peru (*n* = 6), Germany (*n* = 5), Sweden (*n* = 4), Italy (*n* = 2), Mexico (*n* = 2), Austria (*n* = 1), Argentina (*n* = 1), Brazil (*n* = 1), Belgium (*n* = 1), Greece (*n* = 1), and Switzerland (*n* = 1). The 342 *G*. *parasuis* isolates were collected from 14 provincial-level administrative divisions (PLADs) in China, including 193 and 149 isolates collected from diseased and healthy pigs, respectively (Fig. 1B). Upon analysis, the final genome assemblies of 764 isolates ranged in size from 2,064,598 to 2,570,730 bp, with an average GC content of 39.46%–40.39%, which was comparable to the publicly available draft and complete genomes of *G. parasuis*.

### Multilocus sequence type diversity and patterns

**Fig 2:**
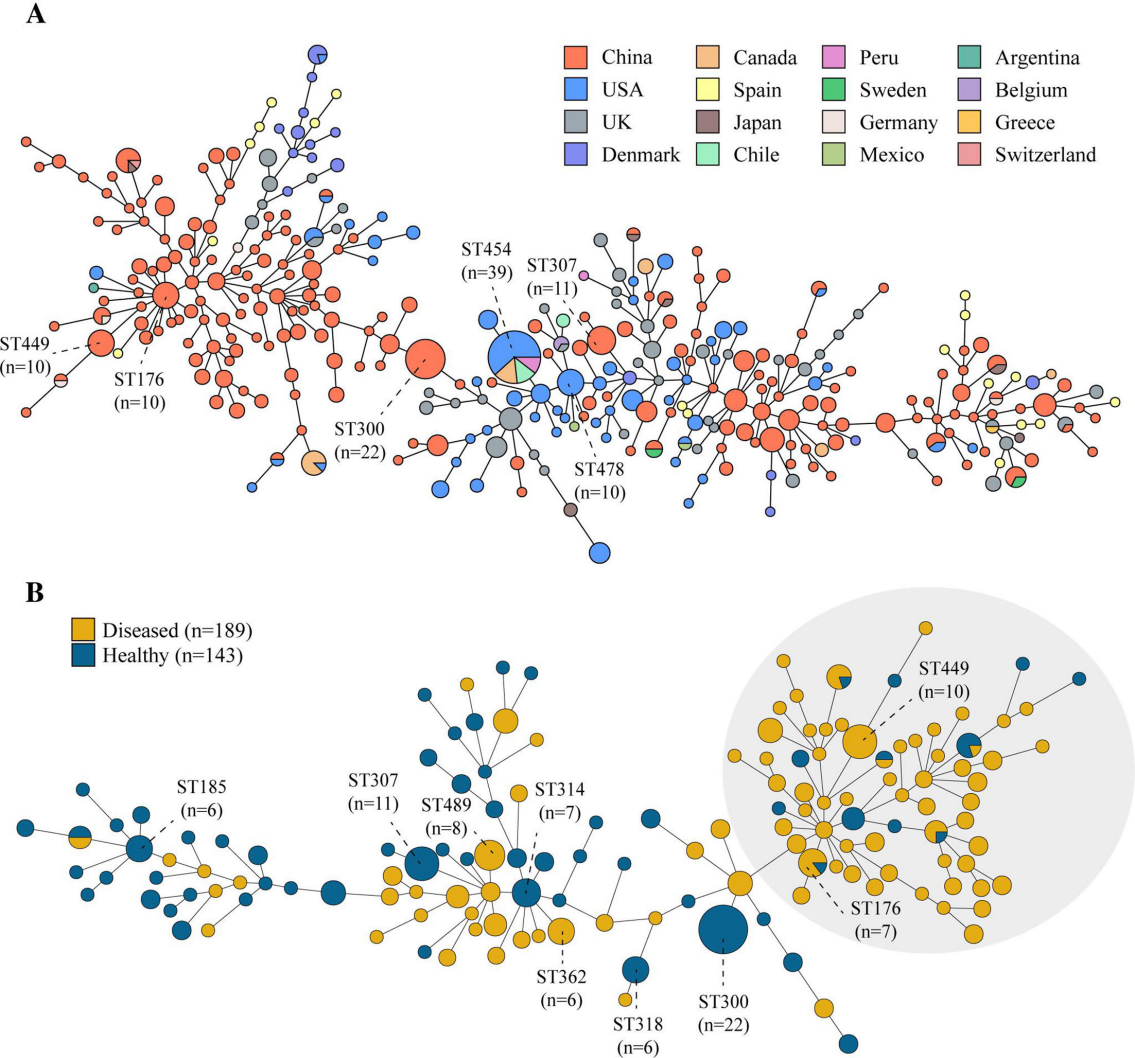
Minimum spanning tree of the whole data set based on multilocus sequence typing. (**A**) Minimum spanning tree of 709 *G*. *parasuis* isolates. Different colors indicate the geographic location of the isolates. (**B**) Minimum spanning tree of 332 *G*. *parasuis* isolates. The source of isolates is indicated using yellow and blue. The region in light gray (right branch) comprising most strains originated from diseased pigs.

Owing to incomplete alleles or the presence of new alleles, 55 isolates were excluded from the construction of the minimum spanning tree (MST; Fig. 2A). The 709 isolates were assigned to 334 different STs, with ST454 being the most frequent (5.10%, 39/764) and comprising isolates from the USA, Canada, Chile, and Peru. More than half of the STs (57.78%, 193/334) were represented by a single isolate. The 332 *G*. *parasuis* isolates we collected were assigned to 147 different STs, 44 of which were novel. More than half of the isolates from diseased pigs (59.79%, 113/189) were concentrated in the right branch, and the predominant STs were ST449 (5.29%, 10/189) and ST176 (3.70%, 7/189). ST300, which was found only in isolates from healthy pigs, was the most prevalent ST, with a prevalence rate of 15.38% (22/143), followed by ST307 (7.69%, 11/143), ST314 (4.90%, 7/143), ST318 (4.20%, 6/143), and ST185 (4.20%, 6/143). All the above ST patterns in healthy pigs were located on the left branch (Fig. 2B).

### Serovar profiling

**Fig 3:**
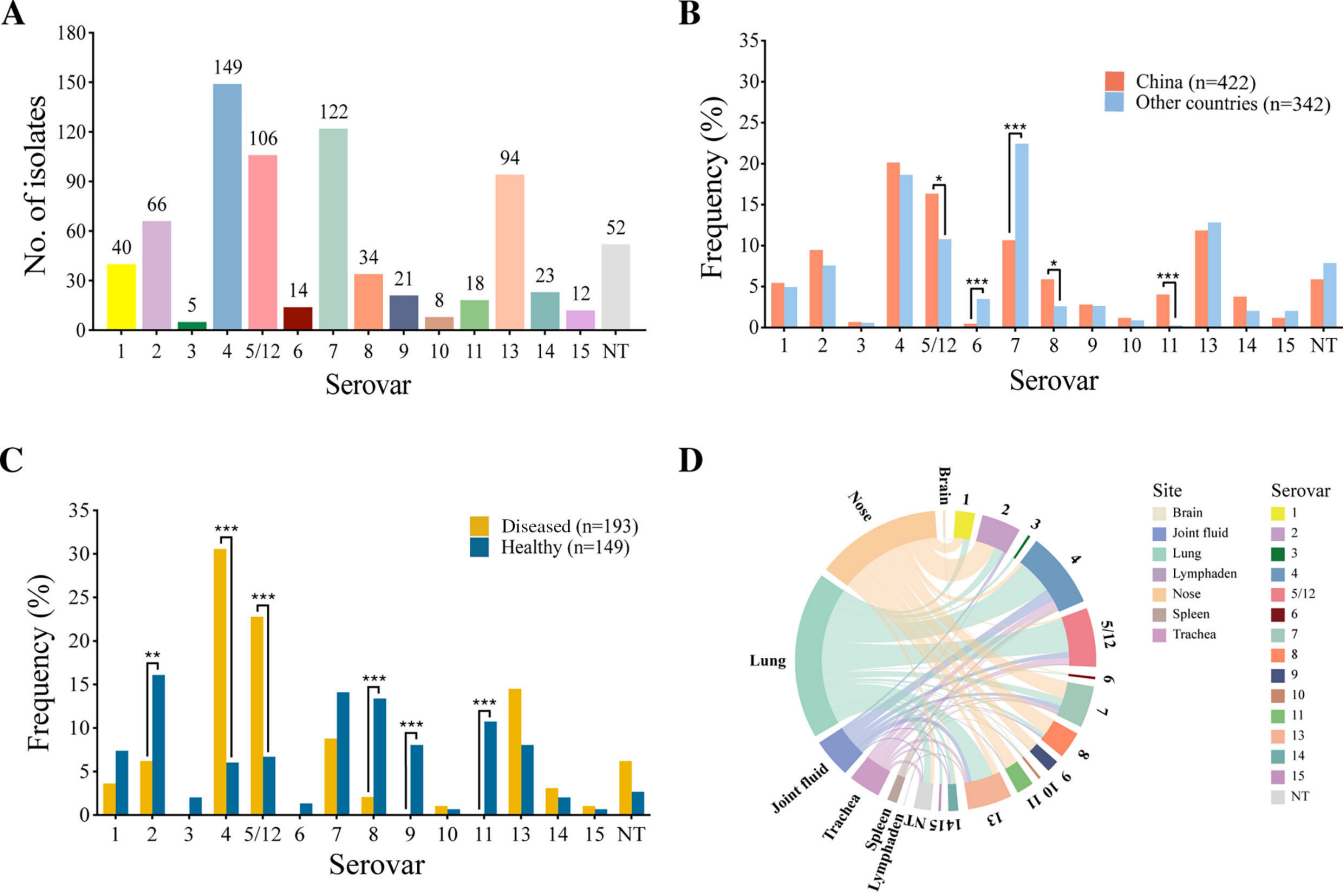
Serovar distribution of 764 *G*. *parasuis* isolates based on molecular serotyping. (**A**) Numbers of *G. parasuis* isolates of different serovars. (**B and C**) Serovar distribution of *G. parasuis* isolates in different countries (**B**) and from different sources (**C**). Statistically significant values are indicated by asterisks. *P < 0.05, **P < 0.01, ***P < 0.001. (**D**) The relationship between serovars and sampling sites (*n* = 342). The serovars and sampling sites are indicated by different colors.

The 764 isolates were allocated to 15 distinct serovars and 52 NT strains based on *in silico* serovar prediction. Serovar 4 (19.50%, 149/764) was the most frequently detected, followed by serovars 7 (15.97%, 122/764), 5/12 (13.87%, 106/764), and 13 (12.30%, 94/764). Serovars 3 and 10 were only present in a small number of isolates, encompassing 1.70% of the isolates (Fig. 3A). The detection frequencies of serovars 5/12, 8, and 11 in China were significantly higher than those in other countries (*P* < 0.05) (Fig. 3B). In contrast, the detection frequency of serovars 6 and 7 in China was significantly lower than that in the other countries (*P* < 0.001). The prevalence of different serovars varied between diseased and healthy pigs. The detection frequencies of serovars 4 (30.57% vs 6.04%) and 5/12 (22.80% vs 6.71%) in diseased pigs were significantly higher than those in healthy pigs (*P* < 0.01). Conversely, the detection frequencies of serovars 2 (16.11% vs 6.22%), 8 (13.42% vs 2.07%), 11 (10.74% vs 0%), and 9 (8.05% vs 0%) were significantly higher in healthy pigs than those in diseased pigs (*P* < 0.05). Strains of serovars 3, 6, 9, and 11 were present only in healthy pigs (Fig. 3C). The serovar distribution of strains isolated from different sites in our collection (*n* = 342) is shown in Fig. 3D. All serovars were isolated from the lungs, with a relatively higher detection rate for serovars 4, 5/12, and 13, and most serovars were detected from the nose, apart from serovars 3 and 15, with a relatively higher detection rate for serovars 2, 7, 8, and 11. Serovars 4, 13, 5/12, and 7 were associated with multiple sampling sites (*n* ≥ 5), whereas serovars 3 and 15 were detected only from the lungs.

### Phylogenetic analysis

**Fig 4:**
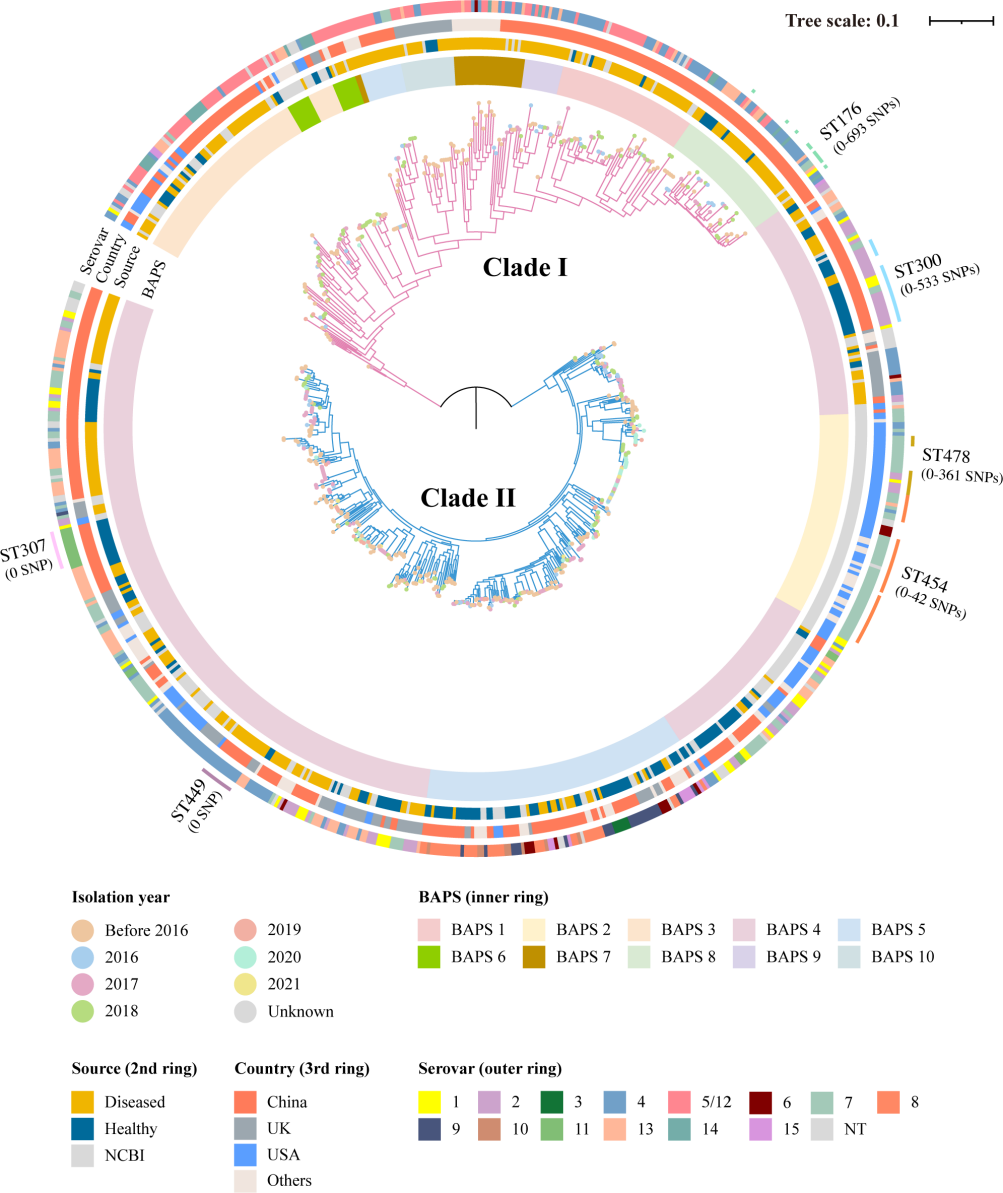
Phylogenetic tree of 764 *G*. *parasuis* isolates based on core-genome SNPs. SH0165 is used as a reference. The collection dates are indicated by different colored circles at the end of the node branches. The annotation rings surrounding the tree, from inside to outside, represent the BAPS (inner ring), source (second ring), country (third ring), and serovar (outer ring), respectively. Clades are represented by different colors, with clade I in pink and clade II in blue.

To determine the evolutionary relationships between the 764 *G*. *parasuis* isolates originated from our collection and the database, a maximum likelihood phylogenetic tree based on single-nucleotide polymorphism (SNP) was constructed using the complete genome of SH0165 (GenBank accession number: CP001321.1) as a reference. The phylogenetic tree encompassed two divergent clades associated with the source, isolation year, country, and serovar (Fig. 4). Isolates from clade II (69.11%) were more prevalent than those from clade I (30.89%). The isolates collected in 2019–2021 and those originated from the USA and UK predominantly belonged to clade II, with proportions of 93.52%, 85.50%, and 78.43%, respectively. The China isolates were found in equal proportions in both clades. The proportion of strains isolated from diseased pigs in the two clades was comparable, whereas a higher proportion of strains isolated from healthy pigs was observed in clade II (85.19%) than that in clade I (14.81%). All strains of serovar 14 and 91.51% strains of serovar 5/12 were found to be situated within clade I, whereas serovars 2, 3, 6, and 8–11 were only detected in clade II. Serovar 7 was mostly found in clade II. The 764 *G*. *parasuis* isolates were grouped into 10 Bayesian analysis of population structure (BAPS) groups according to a Bayesian model-based population structure analysis, with BAPS 1, 3, and 6–10 found in clade I, and BAPS 2, 4, and 5 identified in clade II. BAPS 4 is the predominant group, representing 46.73% of the total population. Most strains with the same ST clustered in the same branch and shared the same serovar, including ST449 (serovar 4) and ST307 (serovar 11). Notably, most ST454 strains belonged to serovar 7 (36/39, 92.31%), with limited SNP (0–42) divergences.

### Antimicrobial resistance genes

**Fig 5:**
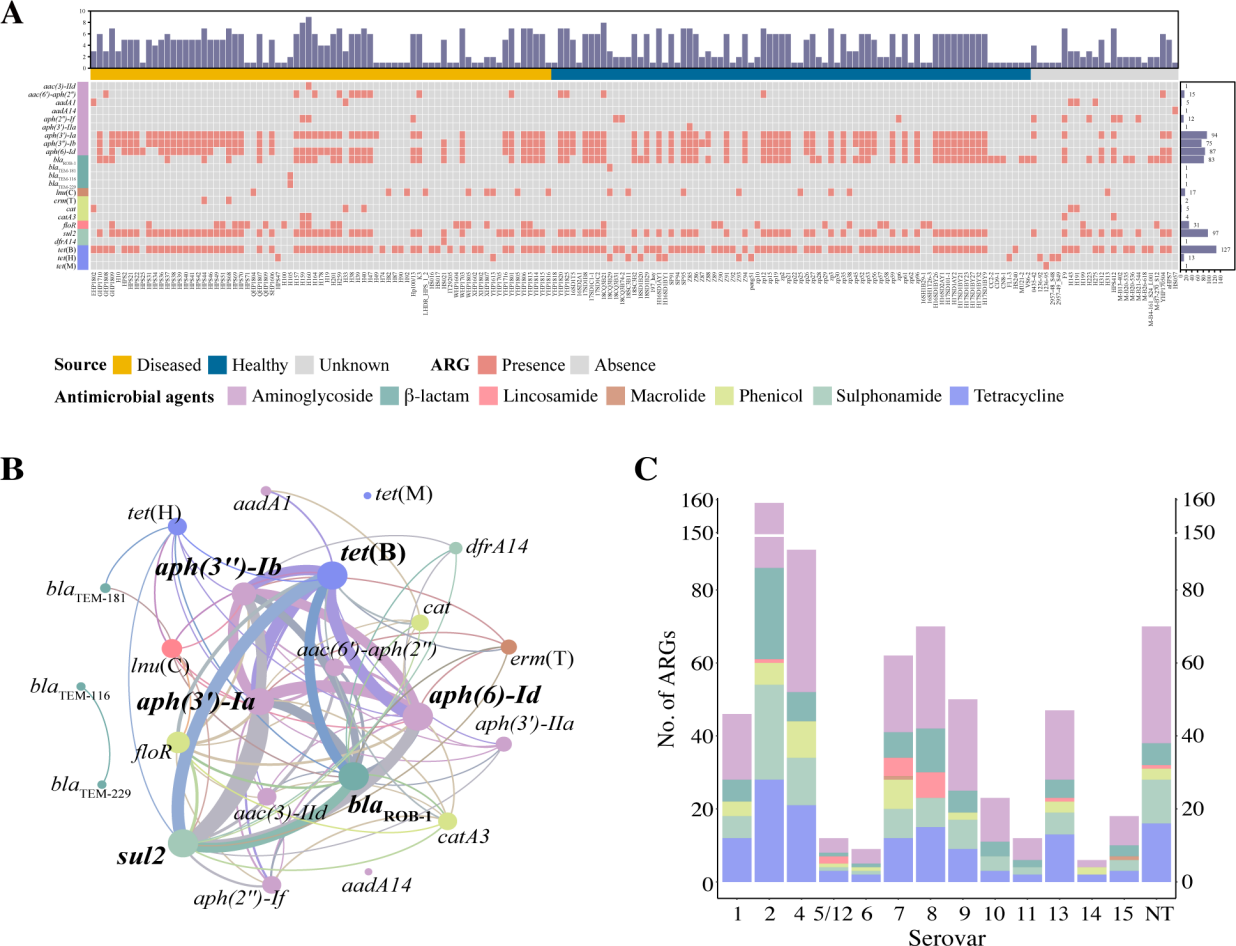
Antimicrobial resistance genes profile of 177 *G*. *parasuis* isolates. (**A**) The presence (pink) and absence (gray) of ARGs in 177 *G*. *parasuis* isolates. The names of 177 isolates are shown on the horizontal axis, and the vertical axis represents 23 ARGs of 7 antibiotic classes. The source of the isolates and the type of antimicrobial agents are indicated by different colors. The length of the top bars denotes the number of ARGs per isolate. The length of the right bars denotes the number of each ARG in 177 isolates. (**B**) The co-occurrence network of ARGs. The nodes are colored according to ARG types. The size of the node is proportional to the number of connections between nodes. (**C**) Composition of the total number of ARGs among different serovars. The ARG types were indicated by different colors.

Based on the ResFinder database, 23 different ARGs that mediated resistance to 7 classes of antibiotics were detected in the 764 *G*. *parasuis* isolates. The positive rate of ARGs was 23.17% (177/764), with the predominant ARG being tetracycline resistance gene *tet*(B), which accounted for 16.62% (127/764), followed by *sul2* (12.70%), *aph(3')-Ia* (12.30%), *aph (6)-Id* (11.39%), *bla*_ROB-1_ (10.86%), and *aph(3'')-Ib* (9.82%). The macrolide resistance gene *erm*(T) (*n* = 2); β-lactam resistance genes *bla*_TEM-181_, *bla*_TEM-116_, and *bla*_TEM-229_ (*n* = 1); and sulfonamide resistance gene *dfrA14* (*n* = 1) were only detected in diseased pigs. On the other hand, the aminoglycoside resistance gene *aph(3')-IIa* (*n* = 1) was detected only in healthy pigs (Fig. 5A). In addition, 102 *G*. *parasuis* isolates carried 3 or more ARGs (Table S1). Figure 5B shows 23 nodes and 90 edges that describe the co-occurrence patterns among different ARGs. Aminoglycoside resistance genes *aph(3')-Ia*, *aph (6)-Id,* and *aph(3')-Ib* were often found together and tended to co-occur with resistance genes of other antibiotics, such as *tet*(B), *sul2,* and *bla*_ROB-1_. These above six genes were also the most frequently detected ARGs. A total of 49 isolates contained all 6 ARGs simultaneously, and Basic Local Alignment Search Tool (BLAST) analysis designated them to 2 types containing the 6 ARGs; however, type II assembled with an additional X ORFs/genes at the start of the loci. It should be noted that this could be an artifact of incomplete sequence data for the type I loci (Fig. S1A). One representative strain (HPS44) of type II, a 23,860-bp multi-resistance region was bracketed by two insert sequence (IS) elements (one intact IS*10*, two intact IS*Apl1,* and two truncated IS*Apl1*) in the same orientation, exhibited high homology to the corresponding region of the ICE*Gpa1804* from *G. parasuis* EHP1804 (No. CP069308), with 99.73% identity at 82.68% coverage.

The average number of ARGs was significantly higher in strains from China than in those from other countries (*P* < 0.05) (Fig. S2A). Furthermore, the detection rate of strains carrying ARGs was substantially higher in healthy pigs than in diseased pigs in our collection (47.65% vs 24.35%). However, there was no significant difference in the average number of ARGs detected between diseased and healthy pigs (*P* = 0.59) (Fig. S2B). The highest number of ARGs was detected in serovar 2 isolates. In contrast, only six and nine ARGs were detected in serovars 14 and 6, respectively (Fig. 5C). No ARGs were available for serovar 3. The remaining serovars listed in the figure contained at least three ARG types, with serovar 7 containing ARGs from all seven antibiotic classes (Fig. S2C). The average number of ARGs in serovar 2 was significantly higher than that in serovars 4, 5/12, 7, 8, and 13 (*P* < 0.05) (Fig. S2D). Notably, ARGs were detected in all strains of ST300 (*n* = 22), ST362 (*n* = 6), and ST185 (*n* = 6); we collected from healthy pigs but not in all isolates of the other predominant STs (Table S2).

### Virulence factors

**Fig 6:**
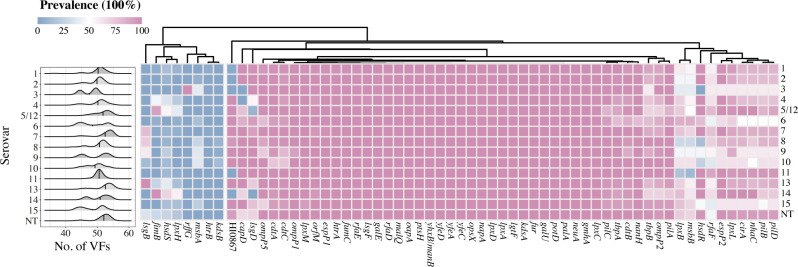
Number and prevalence of VFs among different serovars in the 764 *G*. *parasuis* isolates. Left, distribution of the average number of VFs among different serovars; the median is represented by a black line. Right, the prevalence of VFs within different serovars.

Based on the virulence factor database (VFDB), 24 potential VFs were detected in the 764 *G*. *parasuis* isolates, all of which were associated with lipooligosaccharides (Table S3). Additionally, 37 VFs selected from previous studies were examined using whole-genome sequencing; detailed information on these genes was shown in Table S4. More than half of the VFs were present in almost all isolates, whereas the prevalence of *kdsB*, *rffG*, *htrB,* and *msbA* was extremely low (≤ 4.58%), as shown in Fig. 6. The *capD* gene was absent in serovars 3 and 4, *hsdR* and *lsgD* were present in all serovars except for serovars 3 and 14, respectively. All strains of serovar 13 were positive for *lsgB*, and *rffG* was almost only observed in serovar 3 isolates, with a detection rate of 100%. Furthermore, *fimB* and *hsdS* were both frequently observed among strains of serovars 5/12 and 14. Overall, the average number of VFs was lower in strains from China than in those from other countries (*P* < 0.001) (Fig. S3A); meanwhile, no significant difference in the average number of VFs was observed between the diseased and healthy pigs in our collection (Fig. S3B). The average number of VFs in the strains of serovars 7 and 13 was significantly higher than that in most other serovars (Fig. S3C). In contrast, strains of serovars 3 and 6 had significantly lower numbers of VFs than most other serovars. Among the 15 VFs that were not present in all of the isolates from our collection, the frequencies of *lpxB*, *hsdS*, *fimB*, HI0867, and *hsdR* in diseased pigs were significantly higher than those in healthy pigs, whereas the frequencies of *capD*, *lsgD,* and *lsgB* were significantly lower (Fig. S3D).

## DISCUSSION

The multilocus sequence typing (MLST) scheme serves as an essential tool in delineating the population structures and genetic diversity of *G. parasuis*, with the corresponding MLST database experiencing significant enrichment and expansion in recent years. Among the 764 isolates collected in this study, 709 isolates were assigned to 334 different STs by MLST, with 25.92% of the isolates possessing unique STs. Additionally, we identified 93 new STs, of which 44 were uniquely identified within our collection. This has enriched the MLST database for *G. parasuis*, highlighting the wide distribution and the very diverse nature of *G. parasuis* strains worldwide. No predominant ST was detected, and the highest frequencies of ST in China and other countries were 3.10% (ST300, 22/709) and 5.50% (ST454, 39/709), respectively, in agreement with a previous report (30). Most strains of the same STs tended to come from the same farm, which could be explained to a certain degree by geographic clonal dissemination, which facilitates the spread of *G. parasuis* and causes disease. In addition, some strains of the same ST appeared to have a wider distribution, as some STs could be detected in different years or geographical regions (Fig. 4).

The determination of *G. parasuis* serovars is critical for developing surveillance and vaccination strategies to prevent the disease. In this study, *in silico* serovar prediction based on the analysis of capsule loci was implemented, as it is considered the new gold standard for serotyping (12). The 764 isolates were allocated to 15 distinct serovars, and 52 isolates could not be assigned to any known serovar, suggesting that a wide range of serovars is prevalent in the swine industry worldwide. The number of NT isolates, with a proportion of 6.81%, decreased compared to the previous report, and it would be acceptable from a diagnostic point of view (13–15, 31). The 6.81% NT strains in this analysis are due to the nature of the incomplete genomes used in this study as well as mutations, deletions, and/or unknown sequences (no significant similarity sequence) in the capsule locus and the BLASTn cutoffs set for this analysis. These strains may indeed be typeable using the available methods in the laboratory to accompany these results (32). Besides, the serovar of an isolate cannot be considered a stable marker of virulence, as isolates in the same serovar may exhibit different virulence levels. The overall distribution of *G. parasuis* serovars has largely remained stable over the past few decades. Notably, serovar 7 has gradually become one of the dominant serovars worldwide and has previously been reported to be associated with Glässer’s disease (33, 34). Nevertheless, serotyping remains a valuable tool for the epidemiological analysis of *G. parasuis*. It should be integrated with the virulence testing in pigs, facilitating the implementation of more adequate control strategies in the clinic. Serovars 4 and 5/12 were strongly present in diseased pigs from our collection, whereas, in healthy pigs, serovars 2, 8, 9, and 11 appeared to be more frequent. The presence of the virulent serovars 2 and 8 in healthy pigs without disease manifestation could be due to pre-existing immunity in the pigs, which may obscure the actual virulence of the strain. Moreover, serovars 3, 6, 9, and 11 were only detected in healthy pigs in our collection, indicating the limited virulence of these serovars (2, 10). There is a risk of isolate misclassification when assessing the virulence of strains from diseased or healthy pigs (35). For instance, isolates from healthy animals may be mistakenly deemed less virulent, while environmental or host factors may indeed be the reason for absence of disease. The sampling site might be related to the virulence of the strains, as isolates of serovars 4, 5/12, and 13 recovered from nearly all sampling sites may cause systemic infection under certain circumstances, especially in diseased pigs (15). In addition, three strains of serovars 1 and 13 were isolated from the brains of diseased pigs (Fig. 3D), which strongly suggests that these two serovars are highly virulent. While, an alternative explanation could be that there was an undetected co-infection with additional pathogens in these cases. Considering the coexistence of both non-virulent and virulent isolates in the nasal cavity, determining the virulence of isolates based on sampling site is likely to result in some inaccuracies.

The phylogeny and population structure of *G. parasuis* strains comprised two distinct clades in this study, which aligns with previous findings (1, 30, 36, 37). The two clades, sharing a common ancestor, are estimated to have diverged from 1852 to 1878 (Fig. S4). Our time-calibrated phylogeny revealed that the isolate’s location in the phylogenetic tree is correlated with its date of isolation. Both clades showed high genetic diversity, as isolates from different sources, isolation year, countries, and serovars were found throughout the phylogenetic tree and BAPS populations. Both clades contained isolates from China and the other countries, indicating a possibility of geographical migration of such strains between different countries. Nevertheless, considering the pig industry’s global expanse, with extensive cross-border movement of stock, it is anticipated that geographical separation would exert minimal influence on the population structure. Neither phylogenetic clade displayed a clear association with disease due to the lack of detailed host information, and both clades had mixed results with regard to serovars and health status of the source pig.

Antibiotics are extensively used to prevent and control infections caused by *G. parasuis*. However, the irrational use has led to increased antimicrobial resistance, limiting therapeutic choices. In the current study, we identified 23 ARGs, most of which were identified in previous studies (37–39). The prevalence of these genes in *G. parasuis* is generally higher in China than that in other countries, which may be related to suboptimal drug use in breeding farms in China. We previously hypothesized that strains of serovars considered as non-virulent might carry more ARGs, while a discernible correlation between serovars and ARGs is not evident, as higher average number of ARGs were observed predominantly in strains of serovars 2 and 9. Tetracycline efflux pump encoding gene *tet*(B) was the most prevalent ARG identified in this study, which aligns with the fact that strains from our collection exhibited high MIC for oxytetracycline (≥32 µg/mL) and tetracycline (≥4 µg/mL) (Table S5). Dihydropteroate synthase encoding gene *sul2* is involved in sulfonamide resistance and is usually associated with integrative conjugative elements (ICEs), which facilitate the spread of antimicrobial resistance among *G. parasuis* (40, 41). More than half of the strains that carried the *sul2* gene from our collection showed a higher MIC value above 64 µg/mL for sulfamonomethoxine (Table S5); similar results were observed in other studies (39, 42). Nine aminoglycoside O-phosphotransferase encoding genes *aph(3'')-Ib*, *aph(3')-Ia*, *aph (6)-Id*, *aac(6')-aph(2''*), *aac (3)-IId*, *aph(2'')-If*, *aadA1*, *aph(3')-IIa,* and *aadA14* were found in the current study, which accounted for almost half of all ARGs. To date, only two β-lactam resistance genes (*bla*_ROB-1_ and *bla*_TEM_) have been identified in *G. parasuis* (39, 43). Consistently, *bla*_ROB-1_ was widespread among *G. parasuis* isolates we analyzed. The presence of *bla*_TEM-116_, *bla*_TEM-181_, and *bla*_TEM-229_ in *G. parasuis* isolates was first described in this study, and it needs to be further clarified. In addition, the genes *tet*(M) and *dfrA14* identified in an USA strain and a UK strain, respectively, have been rarely reported. Since the first *erm*(T)-carrying plasmid was identified in China in 2013 (38), *erm*(T) has rarely been detected in *G. parasuis* or other gram-negative bacteria. In our study, we found *erm*(T) was present in only two strains, which could not explain the commonly high MIC values of macrolides in almost isolated strains (Table S5). It is likely that *G. parasuis* possesses other resistance mechanisms against macrolides, such as point mutations in 23S rRNA gene at position A2059G, which increases the efflux pump activity mediated by the two-component signal transduction system, CpxRA, or other unknown mechanisms (44, 45).

Interestingly, 49 isolates in our collection carried at least 6 ARGs, including *tet*(B), *bla*_ROB-1_, *sul2*, *aph(3'')-Ib*, *aph (6)-Id,* and *aph(3')-Ia* and two kinds of IS elements (IS*10* and IS*Apl1*). The 49 isolates were designated to 16 different STs, indicating these 6 ARGs spread via not only vertical transmission but also horizontal transfer. Among these, two strains of different serovars and STs both contained a multi-resistance region flanked by IS*10* and IS*Apl1* in the same orientation, which was extremely similar to the corresponding region of the ICE*Gpa1804*. It might be some kind of variant of Tn*6743*, which contributed to the spread of seven ARGs via horizontal transfer (46). The fragment IS*Apl1-bla*_ROB-1_*-*∆IS*Apl1-sul2-aph(3'')-Ib-aph (6)-Id-aph(3')-Ia*-∆IS*Apl1-erm*(T)*-*IS*Apl1* is a novel genetic structure in this study, differing from the published reports (40, 41, 46). The fragment ∆IS*Apl1-sul2-aph(3'')-Ib-aph (6)-Id-aph(3')-Ia*-∆IS*Apl1* was identified on chromosome in *Histophilus somni* originated from cattle in Canada (MN401320.1) (Fig. S1B). Notably, the two ∆IS*Apl1* located on either side of the four ARGs can potentially be assembled into a complete IS*Apl1*, which suggested that they might transfer as a whole, and IS*Apl1* plays a crucial role in the transmission of these four ARGs between different animals. In addition, the 6,695-bp truncated Tn*10* element carrying *tet*(B) in all 49 isolates was almost the same as ICE*Gpa1804* (99% identity at 100% coverage) and similar to ICE*Apl1* originated from *A. pleuropneumoniae* and plasmids, such as pSJO-60984 of swine origin (CP025277.1), pN13-01290_23 of turkey origin (CP012931.1), and pS2122_1 of human origin (CP110658.1) (Fig. S1C) (47, 48). There was an 8-bp “GCTGAATT” fragment downstream of this truncated Tn*10*; interestingly, this fragment was the first 8 bp of the left inverted repeat sequence of IS*Apl1*. It is indicated that IS*Apl1* might recognize the specific locus on Tn*10*, potentially leading to a tandem arrangement of IS*Apl1,* which in turn captures ARGs (46). Tn*10* is widely distributed in various pathogenic bacteria and ICEs, and ∆Tn*10* may be more susceptible to capture ARGs by integration or recombination, thus exhibiting structural variation in different bacteria (49–52). This could be explained by the high prevalence of *tet*(B) in this study. Tetracycline resistance bacteria can be isolated from food, animals, humans, and the environment, and it is associated with conjugative plasmids and transposon to some content (53, 54). There is a probable potential risk for dissemination of *tet*(B) via Tn*10*-like element to other pathogenic bacteria and hosts.

More than half of the potential VFs were conserved in *G. parasuis* in this current study, which may not make them reliable predictors of virulence. The LOS of *G. parasuis* is associated with endotoxicity, serum resistance, and adhesion, and the virulent strains could evade the host’s innate immune system through sialylation of LOS (25, 55, 56). It is worth noting that all VFs identified based on the VFDB in this study were linked to LOS, suggesting that they are likely to play a role in pathogenesis. The sialyltransferase-encoding gene *lsgB* was reported to be predominantly present in systemic isolates but not in any of the nasal isolates of *G. parasu*is (25). However, in this study, the nasal isolates were positive for *lsgB*, a result at odds with previous studies. Moreover, *lsgB* was present in strains of serovars 7–10 and all the strains of serovar 13, which are considered highly virulent. CapD is a pathogenicity-associated determinant of *G. parasuis* that is involved in serum resistance (57). Our results showed that the *capD* gene encoding a polysaccharide biosynthesis protein was absent in all serovar 3 and 4 strains, same as a previous study (58). Group 1 *vtaA* was associated with *G. parasuis* virulent strains, and group 3 *vtaA* was *G. parasuis* species specific (27). Notably, there is a risk in employing group 3 *vtaA* as a diagnostic marker for detecting *G. parasuis*, as not all strains were positive for it in this study (Fig. S5A). We also used the leader sequence of the *vtaA* genes as a diagnostic tool to predict virulence, with 87.83% of the strains identified as virulent (data not shown) (29).

It is generally thought that a single VF may not be critical in initiating clinical infections, and our data did not show a good correlation with the presence of specific VFs and serovars. It is intriguing that the average number of VFs in serovar 7, which is considered as non-virulent, was significantly higher than almost other serovars. Moreover, the high prevalence of serovar 7 in recent years emphasizes the imperative for enhanced attention and warrants further investigation. Besides, the NT strains that exhibited a higher average number of VFs necessitate increased investigation, as it signifies their potential impact on pathogenic mechanisms. It is particularly noticeable that strains of the non-pathogenic serovars 7 and 11 were positive for group 1 *vtaA*, significantly suggesting that serovars 7 and 11 are disease-associated serovars of *G. parasuis* (Fig. S5B). On the contray, the virulence of serovars 8 and 15 may require a reassessment, as it appears that they lack the group 1 *vtaA*, which could impact their pathogenicity. It is premature to formulate definitive conclusions as additional potential virulence factors not examined in this study may also be necessary for disease development. These identified VFs may contribute to the virulence of *G. parasuis*; however, the role of these VFs in virulence is yet to be confirmed. It would be greatly advantageous to combine serovars, VFs, clinical metadata, and experimental analysis to further characterize the virulence of the strains comprehensively.

In conclusion, the present study demonstrated the high genetic diversity of *G. parasuis* isolates worldwide. *G. parasuis* isolates were classified into two genomic clusters, with serovars 4, 7, 5/12, and 13 being the most prevalent. We also revealed that ARGs [*tet*(B), *sul2*, *aph(3')-Ia*, *aph (6)-Id*, *bla*_ROB-1_, and *aph(3'')-Ib*], and a range of potential VFs were widespread across isolates in respect of different sources and serovars. We supposed that the observed resistance phenotype could be explained by the carriage of ARGs, with the exception for macrolides, where alternative resistance mechanisms are likely involved. Our study showed a lack of clear and significant associations between serovars, VFs, and virulence. However, serovar 7, previously classified as non-virulent, now warrants reconsideration due to its elevated prevalence and relatively high average number of potential VFs in this study. Further studies focusing on a larger number of *G. parasuis* isolates worldwide are required to link ARGs and/or other resistance mechanisms to phenotypic resistance, and more virulence-associated genes and factors should be authenticated by laboratory experiments, such as animal pathogenicity studies.

## MATERIALS AND METHODS

### Sample collection and bacteria identification

The samples were collected strictly according to the relevant standard protocols. Different sampling sites, including lung, trachea, spleen, brain, and lymphaden tissues as well as joint fluid, were collected from pigs with clinical signs of Glässer’s disease. Nasal swabs were collected from pigs without clinical signs of Glässer’s disease and placed in centrifuge tubes containing tryptic soy broth supplemented with 25 µg/mL nicotinamide adenine dinucleotide (NAD) and 5% (vol/vol) bovine serum. All the samples were stored and transported appropriately to the handling laboratories using ice packs within 24 h. Bacteria species were identified by 16S diagnostic PCR (59).

### Genome sequencing and draft genome sequence assembly

Strains were grown overnight in tryptic soy agar supplemented with 25 µg/mL NAD and 5% bovine serum at 37°C with 5% CO_2_ for 18–24 h. The strains were scraped from the plates for genomic DNA preparation. Genomic DNA was extracted using the Wizard Genomic DNA Purification Kit (Promega, Beijing, China) following the manufacturer’s instructions. DNA libraries were constructed using the KAPA HyperPrep Kit Illumina platforms (Roche, Basel, Switzerland) following the standard protocols and then sequenced on the Illumina HiSeq X Ten platform (Annoroad, Beijing, China). The reads quality was analyzed with FastQC v0.11.7 (https://github.com/s-andrews/FastQC) and was processed using Trimmomatic v0.36 (https://github.com/timflutre/trimmomatic). Trimmomatic output was used for *de novo* assembly in SPAdes v3.13.1 (https://github.com/ablab/spades). Considering the read size, contigs of less than 500 bp were removed, and a 20-fold cutoff was stabilized following SPAdes guidelines (60). The QUAST pipeline (https://github.com/ablab/quast) was used to address the quality of the *de novo* assemblies. Finally, all the complete genomes/draft assemblies were annotated via Prokka v1.12 (https://github.com/tseemann/prokka).

### Multilocus sequence typing analysis

The Illumina read sets were screened against the seven housekeeping gene loci of *G. parasuis*, including *atpD*, *infB*, *mdh*, *rpoB*, *6pgd*, *g3pd,* and *frdB* via mlst v2.11 (https://github.com/tseemann/mlst). Novel alleles and STs were assigned through the submission of the data to the MLST database (https://pubmlst.org/organisms/glaesserella-parasuis). An MST was generated using BioNumerics v7.6 (Applied Maths NV, Sint-Martens-Latem, Belgium).

### Molecular serotyping

The *in silico* serovar prediction for *G. parasuis* was based on analysis of the capsule loci, and 14 serovar-specific genes were selected according to Howell et al. (12) for discriminating different serovars except for serovar 5 and serovar 12. The serovar-specific gene name was determined by a nucleotide BLASTn interrogation of the NCBI database (https://www.ncbi.nlm.nih.gov/). When more than one specific BLAST match sequence was found in the same isolate, their identities were aligned further by BLASTn to determine whether the same sequence was obtained. Identical sequences were defined with a threshold of >90% nucleotide identity and >80% coverage.

### Phylogenetic analysis

SNP-based phylogenetic analysis was performed using Parsnp v1.7.4 (https://github.com/marbl/parsnp), and the conserved regions were then filtered out with Pandas v2.0.3 using in-house scripts. The best substitution model was selected using ModelFinder (61). A maximum likelihood phylogenetic tree based on the concatenated cgSNP sequences was constructed using RAxML under the GTRGAMMA evolutionary model and visualized using an online tool iTOL (https://itol.embl.de/) (62). Complete genome of SH0165 (GenBank accession No. CP001321.1) was selected as a reference. Bayesian model-based population structures were identified with HierBAPS v6.0 (https://github.com/gtonkinhill/rhierbaps) (63). Molecular clock phylogeny was inferred by assessing the accumulation of genetic changes over time via root-to-tip regression in TreeTime (64).

### Antimicrobial resistance genes and virulence factors analysis

ARGs were analyzed by searching the ResFinder database (http://genepi.food.dtu.dk/resfinder) using the assembled genomes. The co-occurrence network of ARGs was generated by the visualization software Gephi v0.9.7 (https://gephi.org). VFs were searched against virulence factor database (http://www.mgc.ac.cn/VFs/) via SRST2 v0.2.0 (http://katholt.github.io/srst2/), taking *Haemophilus* pathogenic factors as references. To further confirm the presence of virulence-associated genes that were not included in VFDB, 37 virulence-associated genes from published studies were selected as listed in Table S4 (3, 36, 37, 65–71). In addition, isolates were analyzed for three groups of *vtaA* translocator domain as previously described (27). The presence of each virulence gene in the *G. parasuis* strains was determined by using BLASTn (*e*-value = 1e−5) using a threshold of >90% nucleotide identity and >60% coverage. The NCBI database was used to determine the description of gene products. KEGG Orthology and the cellular localizations of these proteins were predicted using KOBAS v3.0 (http://bioinfo.org/kobas/) and PSORTb v3.0.3 (http://www.psort.org), respectively.

### Statistical analyses

The statistical analysis was conducted in IBM SPSS software v26.0 and R v4.2.0. *χ*^2^ and Fisher’s exact test were used to compare the frequency of serovars. Differences in the average number of ARGs and VFs in isolates between different countries, sources (diseased pigs and healthy pigs), and serovars were all assessed by the Wilcoxon test. *P* < 0.05 was considered statistically significant.

## Data Availability

The data sets including 342 assembled genome sequences generated during the current study are available in the GenBank database under the BioProject accession number: PRJNA1000289. The remaining 422 data are derived from NCBI and ENA, with their corresponding accession numbers available for consultation in Table S6.

## SUPPLEMENTAL MATERIAL

Supplemental figures msphere.00231-24-s0001.pdf Fig. S1 to S5.

Legends msphere.00231-24-s0002.docx Supplemental figure and table legends.

Table S1 msphere.00231-24-s0003.xlsx Resistance gene patterns and the number of resistant isolates.

Table S2 msphere.00231-24-s0004.xlsx Isolates harboring ARGs involved in this study.

Table S3 msphere.00231-24-s0005.xlsx Identification of the potential virulence genes based on VFDB.

Table S4 msphere.00231-24-s0006.xlsx List of 37 potential VFs according to the literature.

Table S5 msphere.00231-24-s0007.xlsx MIC values for 295 *G. parasuis* isolates.

Table S6 msphere.00231-24-s0008.xlsx Description of 422 *G. parasuis* samples collected from NCBI and ENA.

ASM does not own the copyrights to Supplemental Material that may be linked to, or accessed through, an article. The authors have granted ASM a non-exclusive, world-wide license to publish the Supplemental Material files. Please contact the corresponding author directly for reuse.
