# Supplementary figures and images for: Genomic insight into the diversity of Glaesserella parasuis isolates from 19 countries
Source: mSphere. 2024 Aug 28;9(9):e00231-24. doi: 10.1128/msphere.00231-24 (PMC11423579; doi:10.1128/msphere.00231-24)

Fig. S1

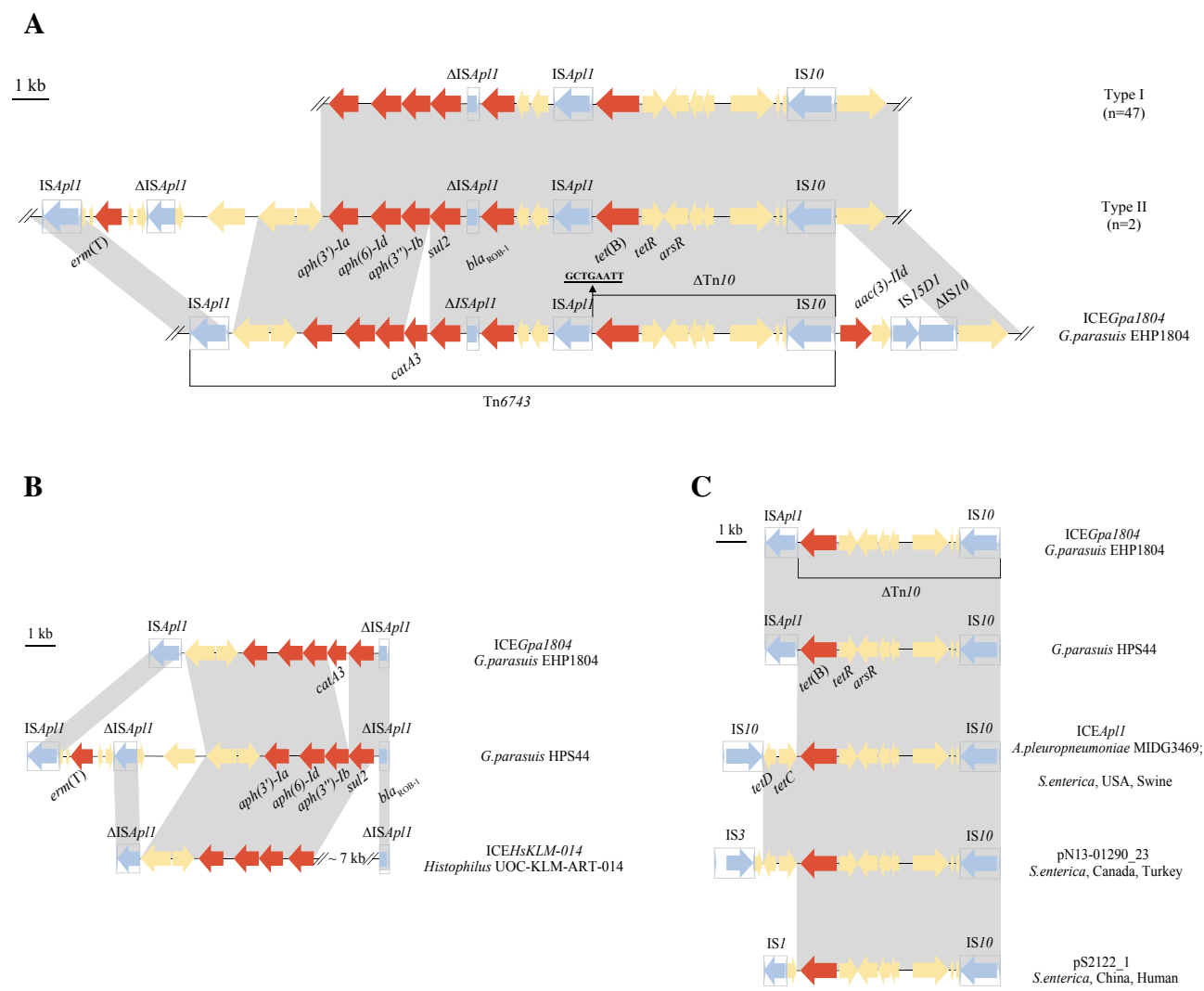

**Fig. S2**

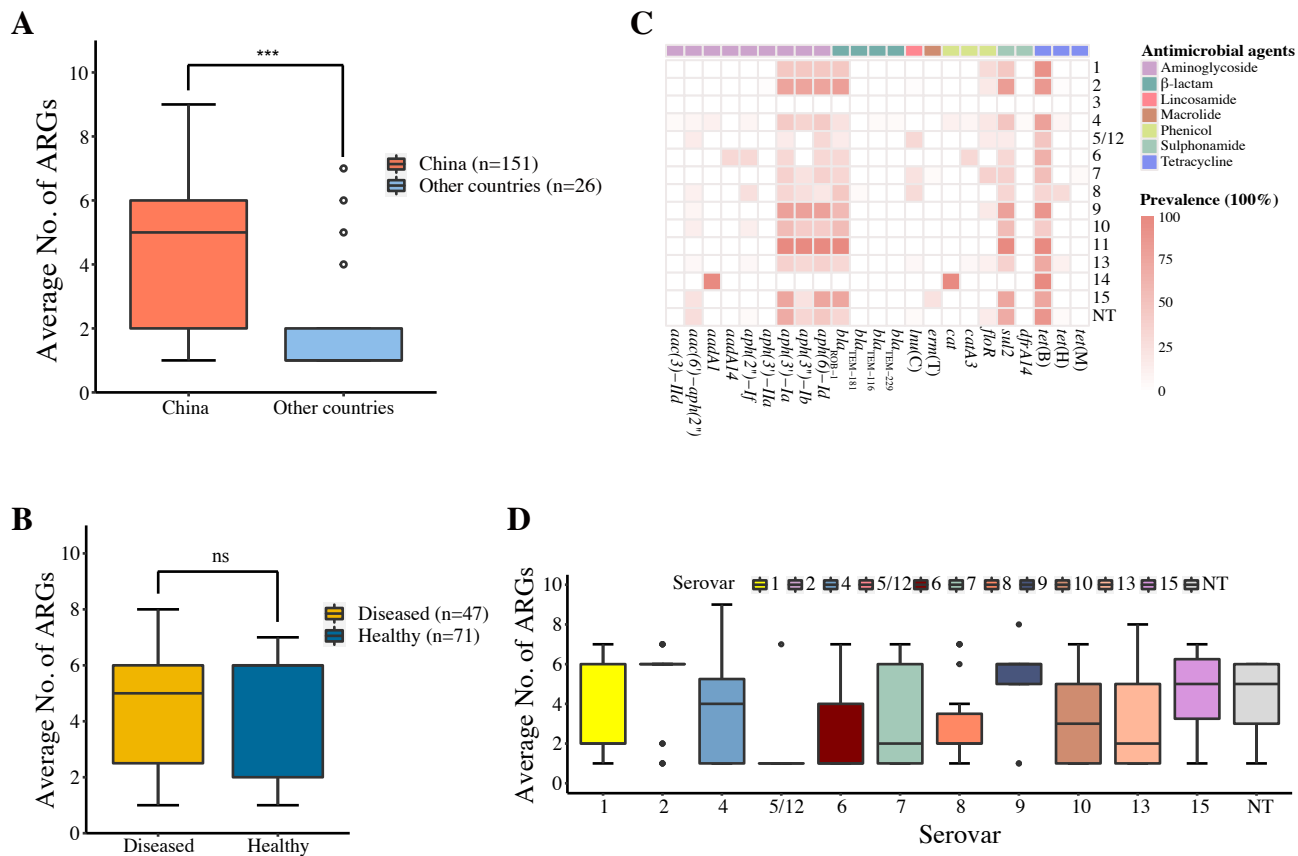

Fig. S3

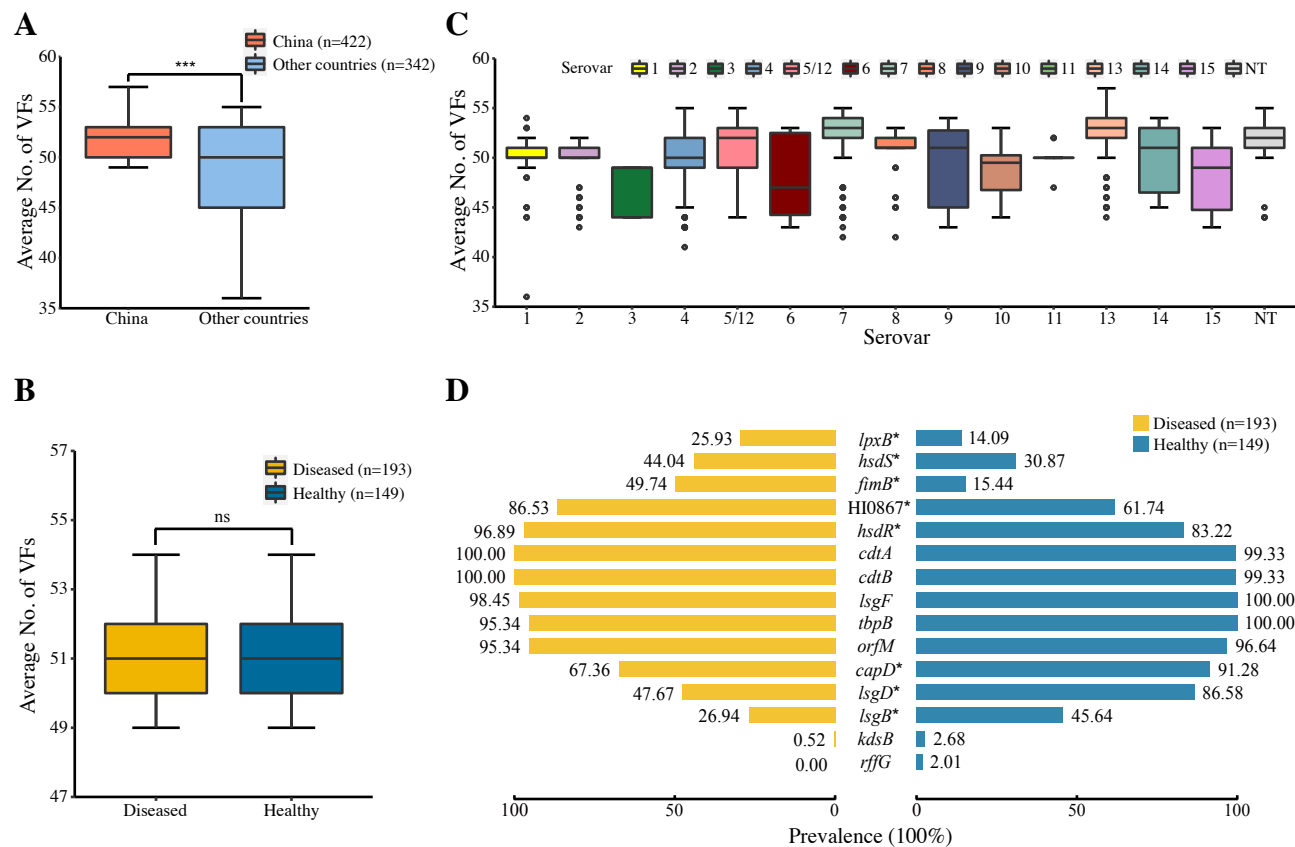

**Fig. S4**

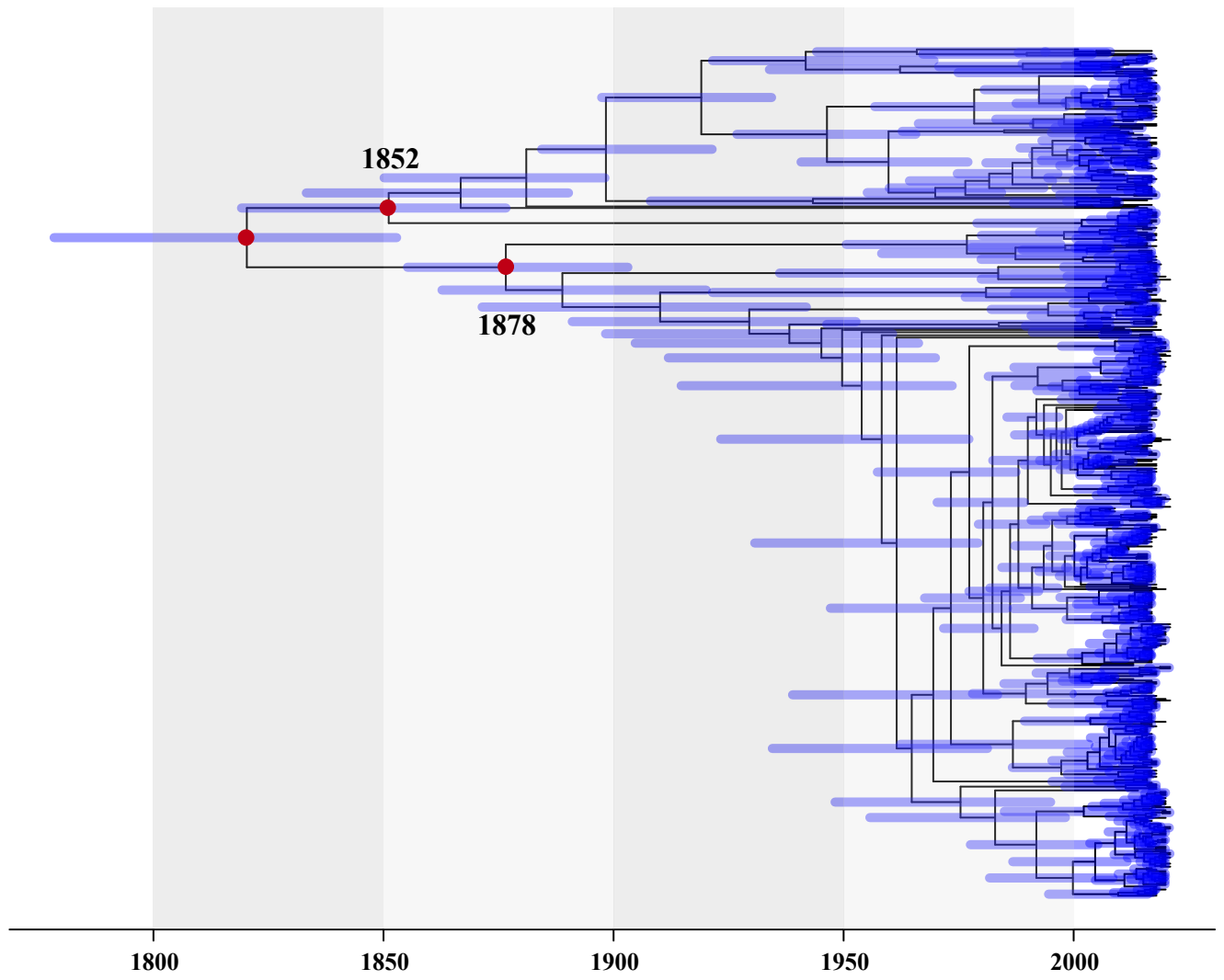

**Fig. S5**

**A**

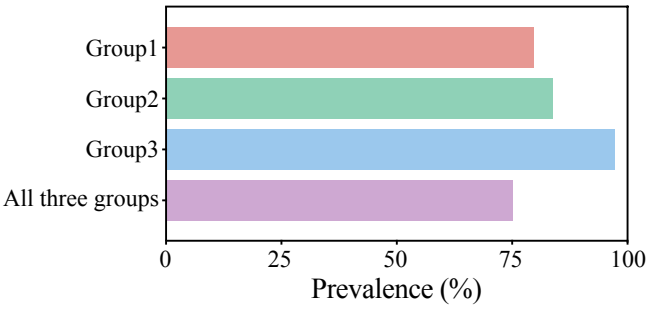

**B**

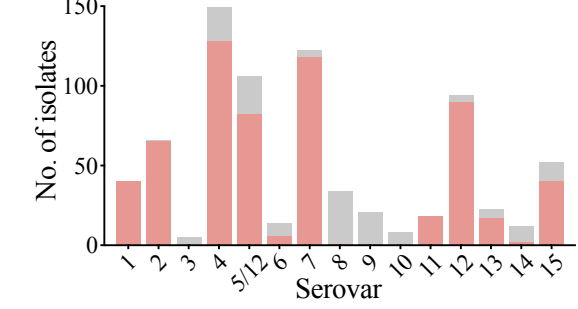

Supplement: Supplemental figures — Fig. S1 to S5. [file msphere.00231-24-s0001.pdf]
