# Supplementary material for: Genomic insight into the diversity of Glaesserella parasuis isolates from 19 countries
Source: mSphere. 2024 Aug 28;9(9):e00231-24. doi: 10.1128/msphere.00231-24 (PMC11423579; doi:10.1128/msphere.00231-24)
Supplement: Legends — Supplemental figure and table legends. [file msphere.00231-24-s0002.docx]

**Supplementary Materials**

**T****able S1** Resistance gene patterns and the number of resistant isolates. ARGs were analyzed by searching the ResFinder Database. 53 resistance gene patterns were involved in this study.

**Table S2** Isolates harboring ARGs involved in this study. 177 isolates harboring ARGs were listed. + means the ARG was present in the corresponding isolate, - means the ARG was absent in the corresponding isolate.

**Table S3** Identification of the potential virulence genes based on VFDB. VFs were searched against VFDB via SRST2 v0.2.0, taking *Haemophilus* pathogenic factors as references.

**Table S4** List of 37 potential VFs according to literatures. The locus in the reference genome (SH0165 strain) is indicated. The subcellular localization was identified by PSORTb v3.0.3.

**Table S5** MIC values for 295 *G. parasuis*. MICs of 18 antibiotics against 295 *G. parasuis* in this study. Strains not tested are indicated by -.

**Table S6** Description of 422 *G. parasuis* collected from NCBI and ENA.

**Fig. S1** Genetic characterization of six ARGs. (A) Comparison of the genetic environment of 49 isolates co-harboring six ARGs in our collection and *G. parasuis* EHP1804. (B) Comparison of the genetic environment of the fragment comprising *sul2*, *aph(3'')-Ib*, *aph(6)-Id* and *aph(3')-Ia*. (C) Comparison of the genetic environment of the fragment comprising *tet*(B). Genes are designated by arrows indicating the direction of transcription. Truncated genes are indicated with a ∆. Homologous regions are represented by light gray shading. *G. parasuis* HPS44 is the representative strain in this study.

**Fig. S2** Distribution of ARGs between isolates from different sources and serovars. Comparison of the average number of ARGs between isolates recovered from different countries (n=177) (A), and different sources (n=118) (B). The median is represented by a black line. Statistically significant values (*P* < 0.05) are indicated using asterisks; ns: not significant. (C) Prevalence of ARGs among different serovars (n=177). The type of antimicrobial agents is indicated by different colors. (D) Comparison of the average number of ARGs between isolates of different serovars (n=173, serovars 3, 11 and 14 are not shown in the figure because the number of isolates was less than three).

**Fig. S3** Distribution of VFs between isolates from different sources and serovars. Comparison of the average number of VFs between isolates recovered from different countries (n=764) (A), and different sources (n=342) (B). The median is represented by a black line. (C) Comparison of the average number of VFs between isolates of different serovars (n=764). (D) Comparison of the prevalence of 15 VFs between isolates from different sources (n=342). The numbers next to the bar represent the prevalence of VFs. Statistically significant values are indicated by asterisks.

**Fig. S4** Time-calibrated phylogenetic tree of 556 *G. parasuis* isolates. Sequences used in the analysis were sampled from 2001 to 2021. Common ancestor internal node is highlighted as the red dot. The 95% confidence intervals are represented by blue lines.

**Fig. S5** Prevalence of *vtaA* and the correlation between group 1 *vtaA* and serovars. (A) Prevalence of *vtaA* translocator groups 1-3. Purple bar indicates isolates positive for all three translocator *vtaA* groups. (B) Correlation between group 1 *vtaA* and serovars. Pink bars indicate isolates positive for group 1 *vtaA*, grey bars indicate isolates negative for group 1 *vtaA*.
